# Editorial: Antimicrobial peptides and mRNA therapy: Clinical, Veterinary, and plant pathology perspectives with special attention to combatting MDR pathogens

**DOI:** 10.3389/fmicb.2022.1030874

**Published:** 2022-09-16

**Authors:** András Fodor, Orsolya Méhi, Maurizio Brivio

**Affiliations:** ^1^Department of Genetics, Institute of Biology, Faculty of Natural Sciences, Eötvös University, Budapest, Hungary; ^2^Department of Genetics, University of Szeged, Szeged, Hungary; ^3^Post-Doctoral Researcher Biological Research Centre, Hungarian Academy of Sciences (MTA), Szeged, Hungary; ^4^Laboratory of Comparative Immunology and Parasitology, Department of Theoretical and Applied Sciences, University of Insubria, Varese, Italy

**Keywords:** multidrug resistance (MDR), panresistance, antimicrobial peptide (AMP), AMP-encoding mRNA, *in vitro* transcribed, *in vivo* translated AMP-encoding mRNA, antimicrobial nucleic acid therapy

Antimicrobial multidrug resistance (MDR) of the different types is an enormous challenge of clinical, veterinary, and plant pathogenic significance (Fodor et al., 2020). Antimicrobial peptides (AMPs) (Ötvös and Wade, 2014; Upert et al., 2021) are of great potential against MDR pathogens, because (a) the MDR pathogens perform a high frequency of collateral sensitivity to AMPs (Fodor et al., 2022); (b) the mobility patterns of antibiotic resistance and AMP- resistance genes are different (Lázár et al., 2018). The delivery of an exogenous AMP to the right location of a eukaryotic organism is a crucial point. This explains the setting of the ambitious goal to invite authors of manuscripts on delivering *in vitro* transcribed (exogenous) AMP- into the cell to be protected, in order to translate it to the respective protective AM-peptide in site, following the logic of mRNA-based vaccination (Sahin et al., 2020; Karikó et al., 2021). Unfortunately no manuscript like that was submitted. Instead, we received valuable papers within the larger scope of the RT dealing with perspectives of AMPs solving MDR-related problems.

The Research Topics (RT), comprise two Reviews and two Original Research papers from the field of antimicrobial resistance.

**A Review** (Le et al.) discusses the sources and mechanisms of antimicrobial peptides (AMPs) against staphylococcal species including *Staphylococcus aureus, S. haemolyticus, S. epidermidis*, and *S. saprophyticus*; and forecasts potential chemotherapies against multidrug-resistant methicillin-resistant *S. aureus* (MRSA).

**Another Review** (Xu et al.) provides an “avenue” for research, development, and application of novel antibacterial agents to reduce the adverse effects of antibiotic resistance in food animal farms. Antibiotic-resistant bacteria (ARB) and antibiotic-resistant genes (ARGs) in food animals are currently considered emerging contaminants, which seriously threaten public health globally.

**Original Research Paper** (Sun et al.), deals with the cecropin-4-derived C18 AMP family in fungal infections against *Candida albicans*, non-albicans *Candida* species in extreme low a minimum inhibitory concentration (MIC). Some C18 derivatives proved efficient on clinical isolates of fluconazole (FLZ)-resistant *C. tropicalis* and also superior to FLZ for killing planktonic *C. albicans via* damaging the cell structure, retarding hyphae transition, and inhibiting biofilm formation in the *Galleria mellonella* model. C18 might inhibit *C. albicans via* triggering mitochondrial dysfunction driven by ROS generation and Ca2 + accumulation.

**Original Research Paper** (Wang et al.) is about the evaluation of the antimicrobial efficiency of an ApoE mimetic peptide, COG1410, confirmed to exhibit strong neural protective activity and immunomodulatory function. COG1410 showed antimicrobial activity against pan drug-resistant *Acinetobacter baumannii*, even eliminating large inocula., COG1410 exhibited biofilm inhibition and eradication activity, stability in human plasma, and a low propensity to induce resistance. The mechanism of COG1410 killing was to disrupt the integrity of the cell. The strong synergistic interaction between COG1410 and polymyxin B dramatically reduced the working concentration of COG1410, expanding the safety window of the application in the *C. elegans* infection model and considered as a promising drug-candidate against pandrug-resistant *A. baumannii*.

## Author contributions

AF suggested the idea and conception of initiating that RT, drew the conclusions, wrote the first version of this editorial, which, however, it could not be materialized in the absence of the strong help of MB, who gave the most professional, formatting, and linguistic-grammar helps, and OM. OM proved an excellent editor of the published Research Articles and contributed with invaluable comments. All authors contributed to the article and approved the submitted version.

## Conflict of interest

The authors declare that the research was conducted in the absence of any commercial or financial relationships that could be construed as a potential conflict of interest.

## Publisher's note

All claims expressed in this article are solely those of the authors and do not necessarily represent those of their affiliated organizations, or those of the publisher, the editors and the reviewers. Any product that may be evaluated in this article, or claim that may be made by its manufacturer, is not guaranteed or endorsed by the publisher.
